# EWS-FLI1 low Ewing sarcoma cells demonstrate decreased susceptibility to T-cell-mediated tumor cell apoptosis

**DOI:** 10.18632/oncotarget.26939

**Published:** 2019-05-21

**Authors:** Kelly M. Bailey, Claire M. Julian, Ariel N. Klinghoffer, Heather Bernard, Peter C. Lucas, Linda M. McAllister-Lucas

**Affiliations:** ^1^ Department of Pediatrics, Division of Hematology/Oncology, University of Pittsburgh School of Medicine, Pittsburgh, PA 15224, USA; ^2^ Department of Pathology, University of Pittsburgh School of Medicine, Pittsburgh, PA 15224, USA

**Keywords:** EWS-FLI1, Ewing sarcoma, T-cell, interferon, apoptosis

## Abstract

Metastatic and relapsed Ewing sarcoma typically afflicts the adolescent population and is largely fatal. These bone tumors are most commonly driven by the fusion oncoprotein EWS-FLI1. Ewing tumors demonstrate significant intra-tumoral heterogeneity, and individual tumor cells can express highly variable and dynamic levels of EWS-FLI1. Recent studies revealed that the EWS-FLI1 oncoprotein level (high versus low expression) greatly influences the behavior of Ewing tumor cells. As compared to cells with high EWS-FLI1, Ewing cells in the EWS-FLI1 low state demonstrate an increased propensity for metastasis. In light of these observations, we sought to determine how tumor cell EWS-FLI1 level influences the anti-tumor cell immune response. Since ICAM-1, which can promote tumor cell/T-cell interaction and T-cell activation, is highly expressed on EWS-FLI1 low cells, we hypothesized that EWS-FLI1 low cells would be more susceptible to T-cell mediated tumor cell apoptosis as compared to cells with high EWS-FLI1. Unexpectedly, we found that EWS-FLI1 low cells are more resistant to T-cell mediated apoptosis than EWS-FLI1 high cells. We investigated the potential mechanisms by which EWS-FLI1 level might influence the T-cell anti-tumor response, and discovered that low EWS-FLI1 expression results in upregulation of PD-L1 and PD-L2, both important ligands for the PD-1 immune checkpoint receptor on T-cells. We demonstrated that blocking PD-1 results in a greater increase of T-cell mediated killing of EWS-FLI1 low tumor cells as compared to cells with higher EWS-FLI1 expression. Our studies suggest that Ewing cells in the EWS-FLI1 low expression state may serve as a niche of tumor immune-evasion.

## INTRODUCTION

Ewing sarcoma is a cancer of the bone or soft tissue that is driven by EWS-ETS family member fusion oncoproteins, with the majority of tumors harboring an EWS-FLI1 fusion [1]. Gaining a better understanding of Ewing sarcoma cell behavior and survival is a priority, as the adolescent and young adult patients with this disease have a less than 20% chance of survival if the cancer has metastasized or relapsed [2]. Over recent decades, clinical trials investigating various single agent and combination cytotoxic chemotherapies have not resulted in improved outcomes for patients with advanced Ewing sarcoma. In recent years, immunotherapies have been introduced as treatment options for cancers. Isolated patients with advanced Ewing sarcoma have responded to checkpoint inhibitor therapy such as PD-1 inhibitors [3] and FANG immunotherapy (an autologous, genetically modified tumor-based vaccine approach [4]). An ongoing Phase 3 clinical trial (NCT03495921) continues to examine the impact of FANG/VIGIL immunotherapy (Gradalis, Inc.) for the treatment of advanced Ewing sarcoma. In general, pediatric tumors, including Ewing sarcoma, have demonstrated less immune cell infiltration into the tumor and appear to have a more immuno-suppressive microenvironment than many adult tumors [5–7]. Despite this observation, significant immune cell infiltration and peri-tumoral inflammation are seen at key points during solid tumor therapy, such as at the time of radiation and/or chemotherapy induced tumor cell apoptosis [8, 9]. In addition, solid tumor cells circulating in the vasculature (circulating tumor cells, CTCs) regularly encounter immune cells, and this interaction is thought to influence the anti-tumor immune response [10, 11]. Despite much effort by multiple laboratories, it has not yet been possible to develop a syngeneic/transgenic mouse model of Ewing sarcoma [12], and as a result, very little is known about the Ewing sarcoma immune-microenvironment and the tumoral factors that promote immune evasion.

Recently, the aggressive nature of specific Ewing tumor cell subpopulations has implicated intra-tumoral cell heterogeneity as an important factor that influences Ewing sarcoma progression and metastasis [13–17]. Individual Ewing cells within a tumor can express highly variable levels of the EWS-FLI1 fusion oncoprotein [14], and this variation in EWS-FLI1 expression can significantly alter tumor cell behavior, with cells harboring lower EWS-FLI1 levels showing increased capability to seed the lung [13], upregulate tenascin-C expression and metastasize [16]. Franzetti and colleagues [14] have shown that EWS-FLI1 level in Ewing cells is dynamic and that cells can freely transition from a proliferative state associated with EWS-FLI1 ‘high’ expression to an invasive/aggressive EWS-FLI1 ‘low’ state. Further, the expression profile of the EWS-FLI1 low state includes a dramatic shift in the expression of actin cytoskeletal proteins, specifically proteins involved in Rho mediated cell motility. Given the difference in behavior between cells in the EWS-FLI1 high versus low state, we questioned whether differences in immune-evasive capabilities also exist between these distinct Ewing tumor cell subpopulations.

Under basal conditions, EWS-FLI1 low cells express surface ICAM-1 and EWS-FLI1 high cells do not [14]. Whether differences in ICAM-1 exist between EWS-FLI1 high and low cells under conditions of inflammation, such as cell stimulation by interferon gamma, is not known. ICAM-1, also known as CD54, is a cell transmembrane protein that is commonly expressed on the surface of leukocytes, endothelial cells and some types of cancer cells [18]. Leukocytes express LFA-1, the receptor that binds ICAM-1, and tumor cell expression of ICAM-1 facilitates leukocyte adhesion to tumor cells through ICAM-1/LFA-1 interactions. ICAM-1 expression on tumor cells can also serve as a mechanism by which tumor cells enhance T-cell activation [19–21]. Differences in ICAM-1 expression between Ewing cells with high and low EWS-FLI1 may suggest that these sub-populations of Ewing tumor cells could possess innate differences in their ability to engage T-cells. We hypothesized that since EWS-FLI1 low cells express ICAM-1, these cells would be more capable of T-cell engagement as compared to EWS-FLI1 high cells and as a result, EWS-FLI1 low cells would be more susceptible to T-cell mediated apoptosis. Surprisingly, we found that despite ICAM-1 upregulation, EWS-FLI1 low cells are paradoxically more resistant to T-cell mediated killing. We have identified upregulation of PD-L1 and PD-L2 as likely mechanisms for the enhanced resistance of EWS-FLI1 low cells. Our results suggest that EWS-FLI1 low cell state may promote immune evasion in Ewing tumors.

## RESULTS

### Exposure to T-cells enhances Ewing tumor cell ICAM-1 expression

**Figure 1:**
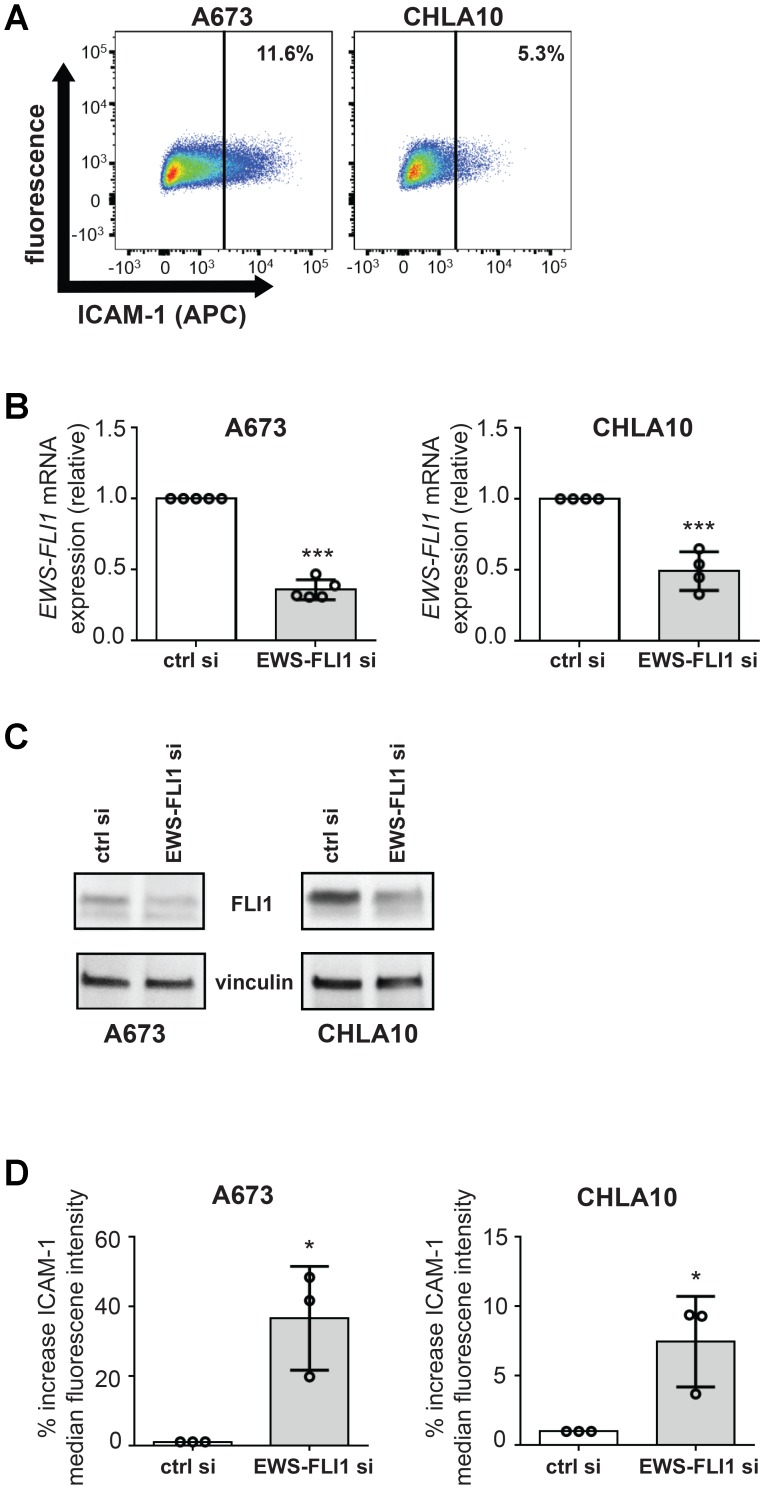
EWS-FLI1 and ICAM-1 expression are inversely correlated under basal conditions in our model system. (**A**) Ewing sarcoma cell lines A673 and CHLA-10 were labeled using an APC-conjugated ICAM-1 antibody and near-IR live/dead stain. Gates were generated to analyze live cell singlets with ICAM-1 staining. % denotes the frequency of ICAM-1+ cells upon analysis of a minimum of 10,000 total events. (**B**) A673 and CHLA10 cells were treated with control or EWS-FLI1 siRNA for 72 hours. Generated cDNA was analyzed for EWS-FLI1 and RPLP0 (control) expression using RT-PCR. Relative expression normalized to control siRNA is graphed. Cells treated with control or EWS-FLI1 siRNA were also either (**C**) lysed and subjected to western blot analysis for FLI1 and a vinculin loading control or (**D**) stained and analyzed by flow cytometry for ICAM-1 surface expression (*n* = 3 per cell line). Error bars reflect SD. Circles on bar graphs in B and D indicate values for individual replicates. Control versus EWS-FLI1 siRNA treated cells were compared using an unpaired *t*-test. ^*^p < 0.05, ^***^p < 0.001.

In order to better understand how the Ewing tumor cell EWS-FLI1 expression level impacts tumor immunity, we first sought to determine if differences exist between the EWS-FLI1 high and low cell response to T-cells. Basal EWS-FLI-1 and ICAM-1 expression are known to be inversely correlated, with EWS-FLI1 low cells expressing significant ICAM-1 [14]. ICAM-1 expressing cells are the minority of cells in both A673 and CHLA-10 cells in culture (Figure 1A). The percentage of ICAM-1 expressing Ewing tumor cells we noted in culture is in line with values reported from prior studies [14]. In order to study EWS-FLI1 high versus low cells, we used an siRNA approach [22, 23]. We were able to validate the inverse relationship of EWS-FLI1 and ICAM-1 expression in the siRNA- based system (Figure 1B–1D), suggesting that cells subjected to siRNA mediated EWS-FLI1 reduction can serve as a model for studying EWS-FLI1 low cells.

**Figure 2:**
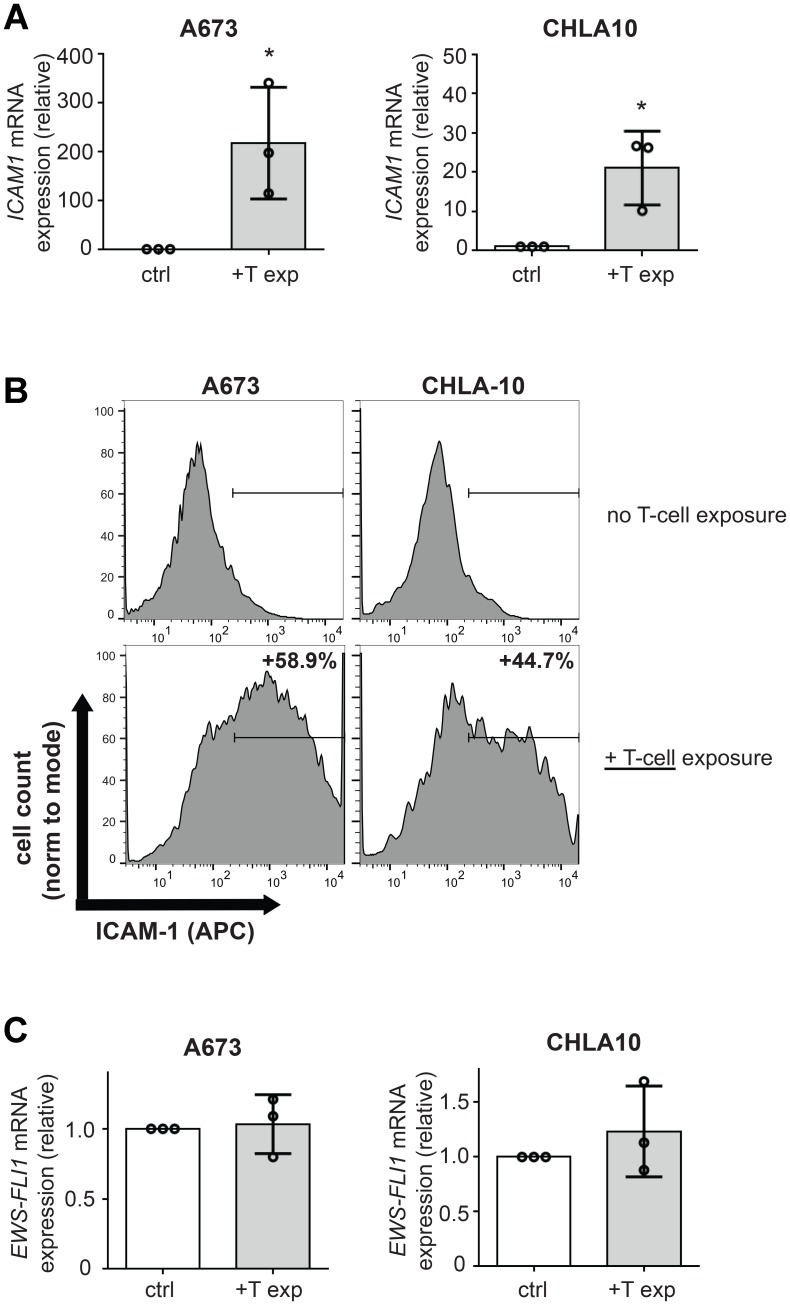
T-cell exposure leads to increased Ewing tumor cell ICAM-1 expression without changing EWS-FLI1 level. A673 or CHLA-10 Ewing tumor cells were co-cultured ± activated T-cells at a ratio of 1 T-cell per 50 tumor cells for 24 hours versus controls (ctrl=no T-cells). Following incubation, T-cells were washed away and tumor cells were analyzed for (**A**) changes in *ICAM1* mRNA expression using RT-PCR (*n* = 3) and (**B**) ICAM-1 surface expression using flow cytometry analysis. Graphs in (B) demonstrate live singlet cell populations. % denotes the frequency of ICAM-1+ cells upon analysis of a minimum of 10,000 total events. (**C**) RNA/cDNA from tumor cells in (A) was also analyzed for changes in EWS-FLI1 expression using RT-PCR (*n* = 3). Error bars represent SD. ^*^p < 0.05. Circles on bar graphs in A and C indicate values for individual replicates.

Since several cancers have been shown to upregulate ICAM-1 expression after exposure to T-cells [24, 25], we tested whether this was also the case for Ewing tumor cells. We co-cultured Ewing cells with activated, random donor T-cells. Following co-culture, T-cells were washed away and Ewing tumor cells were then analyzed for ICAM-1 expression. We observed a dramatic increase in *ICAM1* mRNA and ICAM-1 protein expression in both A673 and CHLA10 cells following T-cell exposure (Figure 2A, 2B). This effect was also confirmed in a third cell line, SK-N-MC (Supplementary Figure 1A, 1B). Interestingly, T-cell exposure-mediated increases in Ewing cell ICAM-1 expression occurred in the absence of any change in *EWS-FLI1* expression level (Figure 2C and Supplementary Figure 1C) suggesting upregulation of ICAM-1 on Ewing cells in response to T-cell exposure occurs via a mechanism that does not necessarily require a lowering of EWS-FLI1 level.

### IFN-γ mediates T-cell induced increases in Ewing tumor cell ICAM-1 expression

**Figure 3:**
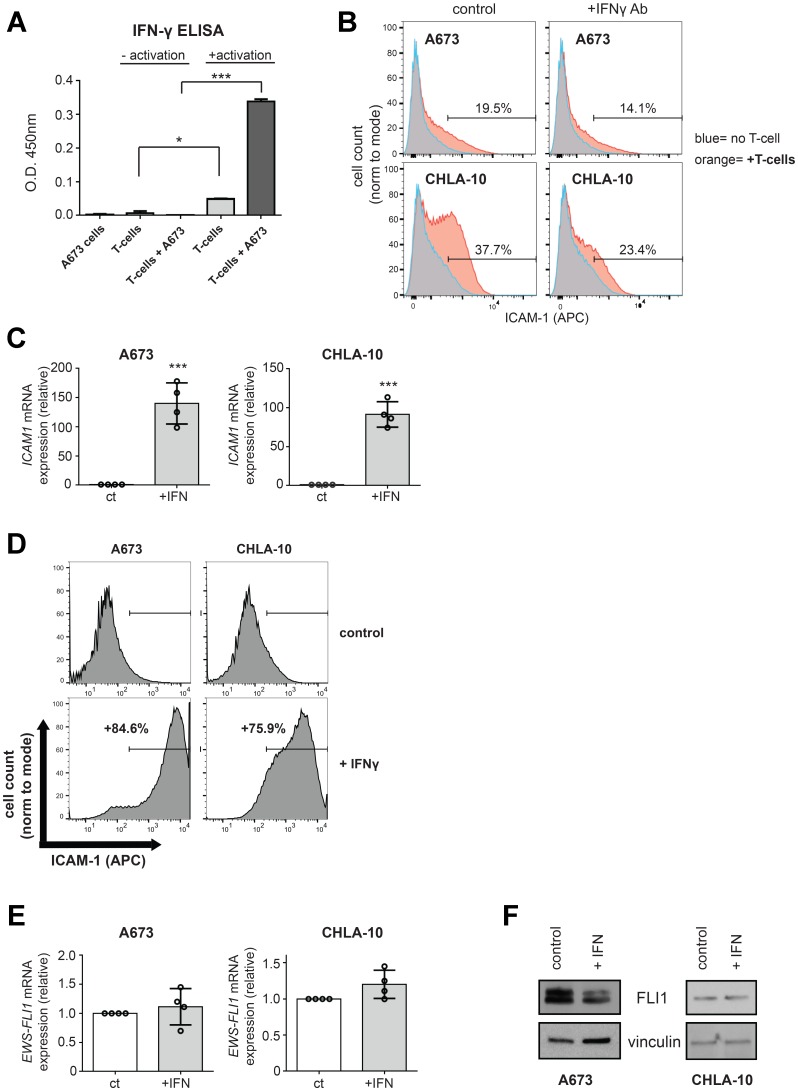
IFN-γ mediated increases in Ewing tumor cell ICAM-1 expression can occur in the absence of changes in EWS-FLI1 expression. (**A**) IFN-γ ELISA was performed on conditioned media from A673 cells alone, T-cells ± activation alone, or co-cultures of unactivated or activated T-cells with A673 Ewing tumor cells. Unactivated/activated T-cell groups were compared using an unpaired *t*-test. ^*^p < 0.05, ^***^p < 0.001. (**B**) A673 (top panels) and CHLA-10 (bottom panels) Ewing tumor cells were treated with IgG control (left panels) or IFN-γ (right panels) in the absence (blue) or presence (orange) of activated T-cells for 5 hours. T-cells were washed away and tumor cells were analyzed for surface ICAM-1 expression by flow cytometry. (**C**, **D**) A673 and CHLA-10 cells were treated with 500 U/mL IFN-γ (+IFN) or vehicle control (ct) for 48 hours followed by RNA isolation and analysis for *ICAM1* expression by RT-PCR (*n* = 4) (**C**) or analysis for surface ICAM-1 by flow cytometry (D). (**E**) RNA/generated cDNA from samples in (C) were also analyzed for changes in EWS-FLI1 expression by RT-PCR. Expression is graphed relative to control (normalized to 1). (**F**) A673 and CHLA-10 cells were treated with vehicle control or IFN-γ for 12 hours. Lysates were analyzed for FLI1 protein expression via western blot. Graphs in B and D demonstrate live, singlet cell populations and % denotes the frequency of ICAM-1+ cells upon analysis of a minimum of 10,000 total events. Circles on bar graphs in C and E indicate values for individual replicates. ct/+IFN cell groups were compared using an unpaired *t*-test. Error bars represent SD. ^***^p < 0.001.

We next sought to determine the mechanism by which T-cells induce ICAM-1 expression in co-cultured Ewing tumor cells. The ability of T-cells to increase ICAM-1 expression on neighboring tumor and stromal cells is thought to be mediated primarily through IFN-γ produced by the T-cells [20, 21, 26]. Using ELISA, we confirmed that the activated human T-cells in our co-culture system do secrete IFN-γ (Figure 3A). To determine if IFN-γ contributes to the increased ICAM-1 expression noted in the Ewing tumor cells following T-cell co-culture, we tested the effect of neutralizing IFN-γ using a blocking antibody. We found that IFN-γ blocking antibody blunts the early T-cell mediated increases in Ewing tumor cell ICAM-1 expression in both A673 and CHLA10 cells (Figure 3B).

We also examined the effect of directly treating Ewing tumor cells with IFN-γ on tumor cell ICAM-1 expression. Ewing cells were treated with recombinant IFN-γ or vehicle control and tumor cell *ICAM1* mRNA and ICAM-1 protein expression was then examined both by RT-PCR and flow cytometry analysis, respectively. Indeed, treatment of Ewing sarcoma cells with recombinant IFN-γ leads to a significant increase in ICAM-1 expression in the majority of cells (Figure 3C, 3D, Supplementary Figure 1D, 1E). Similar to the effect seen with T-cell co-culture, tumor cell treatment with IFN-γ leads to an increase in ICAM-1 expression in the absence of changes in *EWS-FLI1* mRNA expression (Figure 3E, Supplementary Figure 1F). Becuase EWS-FLI1 protein expression level could be effected in the absence of mRNA expression changes, we also performed western blot analysis for EWS-FLI1 (FLI1) protein expression following treatment with vehicle control or IFN-γ for 12 hours. We noted some heterogeneity in the effect on EWS-FLI1 protein expression between cell lines following IFN-γ exposure and conclude that: 1) increases in ICAM-1 expression are not strictly dependent upon a lowering of EWS-FLI1 level during inflammation and 2) in some cell lines, IFN-γ can influence the total level of EWS-FLI1 protein expression, as is noted in A673 cells (Figure 3F).

### Lower EWS-FLI1 level results in augmented response of Ewing tumor cells to IFN-γ

**Figure 4:**
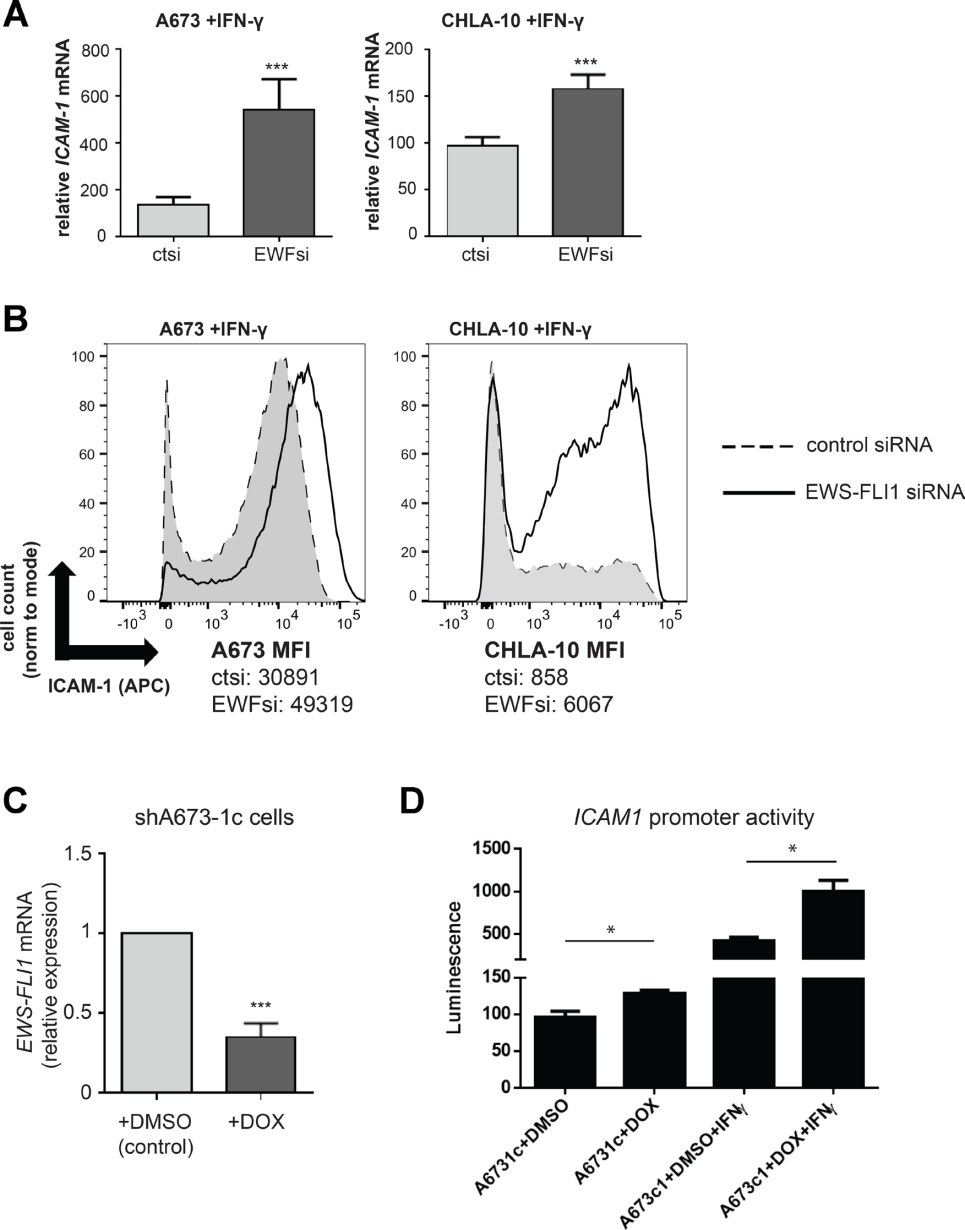
IFN-γ induced ICAM-1 expression is significantly enhanced in EWS-FLI1 ‘low’ Ewing cells. (**A**) A673 (*n* = 4) and CHLA-10 (*n* = 4) cells were treated with control or EWS-FLI1 siRNA for 48 hours, followed by treatment with 500 U/mL IFN-γ for a subsequent 24 hours. Ewing cells were analyzed by RT-PCR analysis for *ICAM-1* and *RPLP0* (control) expression. Graphs represent relative increase in *ICAM1* mRNA expression (values normalized to untreated control). Bars represent SD. Differences in IFN-γ treatment response between ctsi and EWFsi groups for each cell line was compared using an unpaired *t*-test. ^***^p < 0.001. (**B**) Cells were treated with IFN-γ as in (A) followed by analysis of ICAM-1 surface expression by flow cytometry. MFI (median fluorescence intensity) values for both control (ct) and EWS-FLI1 (EWF) siRNA treatment conditions in both the A673 and CHLA10 cell lines are listed. (**C**) shA673-1c cells (harboring shRNA mediated tet-repressible EWS-FLI1 vector) were treated with DMSO vehicle control or 1 microgram/mL doxycycline for 48 hours (*n* = 3). RT-PCR analysis of *EWS-FLI1* and *RPLP0* (control) expression was performed. ^***^p < 0.001. (**D**) An *ICAM1* LightSwitch promoter reporter assay was used to examine differences in promoter activity between shA673-1c cells + DOX (doxycycline) or DMSO control cells in the absence or presence of IFN-γ. In this system, +Dox= EWS-FLI1 ‘low’ expression. Differences in promoter activity between conditions were determined using an unpaired *t*-test. ^*^p < 0.05.

We next asked whether the level of Ewing cell EWS-FLI1 expression impacts the response of Ewing tumor cells to IFN-γ treatment. Specifically, we compared the IFN-γ dependent mediated induction of *ICAM1* mRNA in control versus EWS-FLI1 siRNA treated Ewing cells (Figure 4A). While both cell groups (control and EWS-FLI1 siRNA treated) demonstrated a significant IFN-γ-induced increase in *ICAM1* mRNA expression, the relative induction of *ICAM1* was much greater in cells with low EWS-FLI1 expression as compared to cells with higher EWS-FLI1 expression. We also found that ICAM-1 surface protein expression is significantly greater in IFN-γ treated EWS-FL1 low cells as compared to IFN-γ treated control cells (Figure 4B). We then used an additional and complementary approach to assess the effect of lowering EWS-FL1 level on response to IFN-γ. Specifically, we utilized shA673-1c cells, which harbor shRNA mediated tet-repressible EWS-FLI1 expression [27], and an *ICAM1* LightSwitch promoter reporter system (Figure 4C). While treatment of both the EWS-FLI1 high (DMSO control) and low (+DOX) cells with IFN-γ increases *ICAM1* promoter activity, this increase was significantly larger in cells with low EWS-FLI1 expression (Figure 4D). Taken together, these results demonstrate that cells with lower EWS-FLI1 level more robustly upregulate ICAM-1 following IFN-γ treatment compared to cells with higher EWS-FLI1.

### Despite higher basal and inducible ICAM-1 expression, Ewing cells with lower EWS-FLI1 level are actually more resistant to T-cell mediated apoptosis

**Figure 5:**
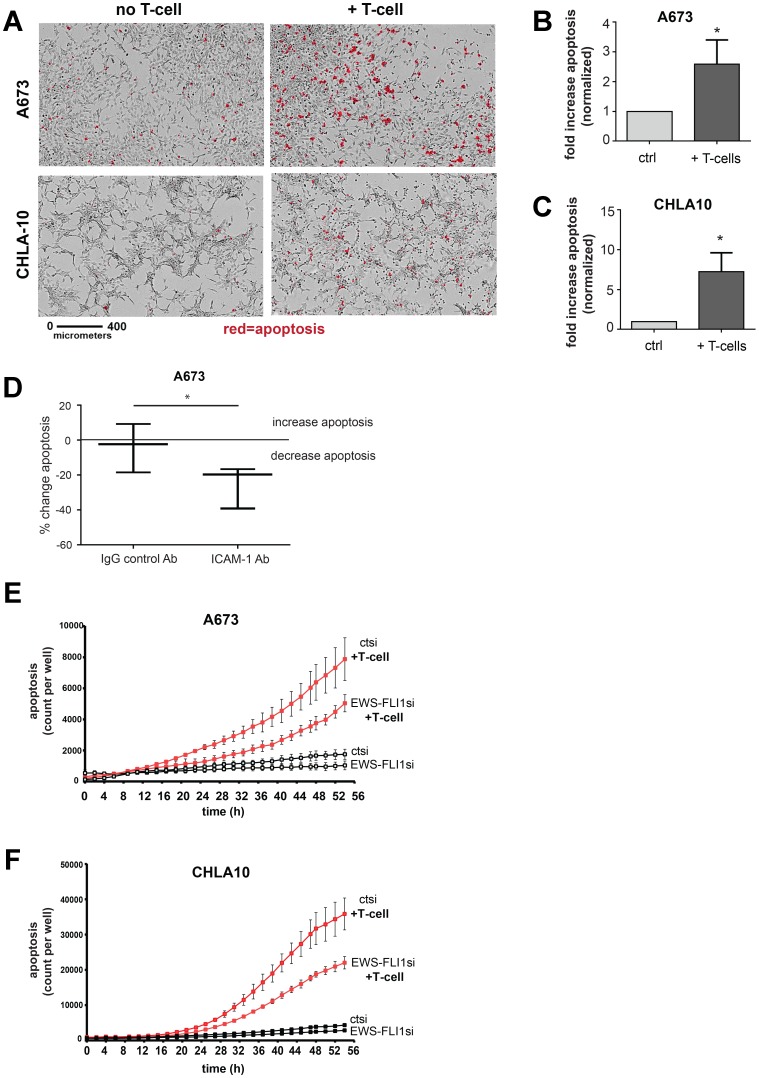
Ewing tumor cells with lower EWS-FLI1 level are more resistant to T-cell mediated apoptosis. (**A**) A673 and CHLA-10 Ewing sarcoma cells were grown alone or in co-culture with activated T-cells for 48 hours and monitored in real time using an IncuCyte system apoptosis assay. Representative images of cells at 36 hours upon real-time monitoring for apoptosis (activated caspase 3/7 activity, red) in tumor cells in culture alone (left panels) or co-cultured with activated T-cells (right panels). The black bar denotes a length of 400 micrometers. (**B–C**) Graphs represent fold increase in apoptosis in A673 and CHLA10 upon exposure to activated T-cells (*n* = 3 per cell line). Differences in apoptosis ± T-cell exposure were determined using an unpaired *t*-test. ^*^p < 0.05. (**D**) A673 Ewing sarcoma cells were left untreated or treated with IgG control or ICAM-1 blocking antibody, co-cultured with activated T-cells and then monitored for caspase 3/7 activity using an IncuCyte apoptosis assay. No T-cell controls were also included (Supplementary Figure 2). Graphs represent the max % change in tumor cell apoptosis with IgG or ICAM-1 antibody treatment compared to no antibody controls (*n* = 3). ^*^p < 0.05. (**E**) A673 (*n* = 4) and (**F**) CHLA-10 (*n* = 3) Ewing sarcoma cells were treated with control (ctsi) or EWS-FLI1 siRNA and placed in the in the absence (black lines) or presence (red lines) of activated T-cells at a ratio of 1:10 (T-cell : tumor cell). Caspase activity was monitored in real time using an IncuCyte apoptosis assay. Error bars represent SD.

We have thus far established that: 1) IFN-γ induces ICAM-1 expression in Ewing sarcoma cells and that 2) Ewing cell EWS-FLI1 level dictates the magnitude of ICAM-1 transcriptional response to IFN-γ, with greater IFN-γ mediated ICAM-1 induction in cells with lower EWS-FLI1. We next wanted to investigate the influence of Ewing cell EWS-FLI1 level on T-cell mediated Ewing cell apoptosis. First, we demonstrate that co-culturing Ewing tumor cells with activated T-cells results in tumor cell apoptosis (Figure 5A–5C). In order to determine how Ewing cell surface ICAM-1 expression impacts T-cell mediated tumor cell apoptosis, we next treated A673 Ewing sarcoma cells with either an IgG control or ICAM-1 blocking antibody, co-cultured with activated T-cells and assessed for apoptosis using an IncuCyte apoptosis assay. As predicted, we found that blocking tumor cell ICAM-1 results in decreased T-cell mediated Ewing cell apoptosis compared to control (Figure 5D). No difference in apoptosis was noted between ICAM-1 Ab versus IgG Ab or no antibody controls in the absence of T-cells (Supplementary Figure 2).

We next wanted to determine the impact of EWS-FLI1 level on T-cell mediated apoptosis. We predicted that EWS-FLI1 ‘low’ cells would be more susceptible to T-cell mediated killing compared to EWS-FLI1 ‘high’ cells given that: 1) EWS-FLI1 ‘low’ cells demonstrate greater basal and IFN-γ inducible ICAM-1 expression, 2) tumor cell ICAM-1 has been shown to enhance T-cell activation in multiple cancers [19–21] and 3) blocking tumor ICAM-1 reduces T-cell mediated Ewing tumor cell apoptosis in our system. We compared rates of apoptosis in control and EWS-FLI1 siRNA treated Ewing sarcoma cells ± T-cells. No appreciable difference in tumor cell apoptosis was observed in the absence of T-cells for either control or EWS-FLI1siRNA treated cells (Figure 5E, 5F). Differences in cell proliferation between control and EWS-FLI1 siRNA treated cells were negligible at the level of EWS-FLI1 low expression used for these experiments. Unexpectedly, we found that despite significantly higher levels of basal and inducible surface ICAM-1 expression, EWS-FLI1 siRNA treated (low) cells do not demonstrate enhanced T-cell mediated tumor cell apoptosis. Rather, EWS-FLI1 low cells demonstrate significantly less T-cell mediated apoptosis as compared to controls (Figure 5E, 5F). Normalization of apoptosis data to cell proliferation did not impact this trend. We further confirmed the cell apoptosis results using a third Ewing sarcoma cell line, SK-N-MC (Supplementary Figure 3A). We also utilized the shA673-1c clone harboring a tet-inducible shRNA that targets EWS-FLI1 (see Figure 4). Like A673, CHLA-10 and SK-N-MC cells treated with EWS-FLI1 siRNA, the shA673-1c cells treated with doxycycline (EWS-FLI1 low) also demonstrate less T-cell mediated apoptosis compared to control cells (Supplementary Figure 3B).

Since tumor cell secretion of soluble ICAM-1, if present, could diminish direct T-cell:tumor cell interaction and thus decrease T-cell mediated tumor cell apoptosis [28, 29], we tested whether sICAM-1 could be responsible for the decreased T-cell mediated apoptosis we observed in the EWS-FLI1 low cells. Upon testing with a sICAM-1 ELISA, we found no sICAM-1 present in the conditioned media from any of our Ewing cell lines or siRNA treated cells (Supplementary Figure 4). These results indicate that Ewing cells with lower EWS-FLI1 express factors that actually dampen the T-cell response, thus overriding the effect of enhanced surface ICAM-1 expression on promoting T-cell interaction and activation.

### EWS-FLI1 ‘low’ cells express more PD-L1 and PD-L2 in response to IFN-γ and blocking PD-1 enhances EWS-FLI1 ‘low’ cell susceptibility to T-cell mediated apoptosis

**Figure 6:**
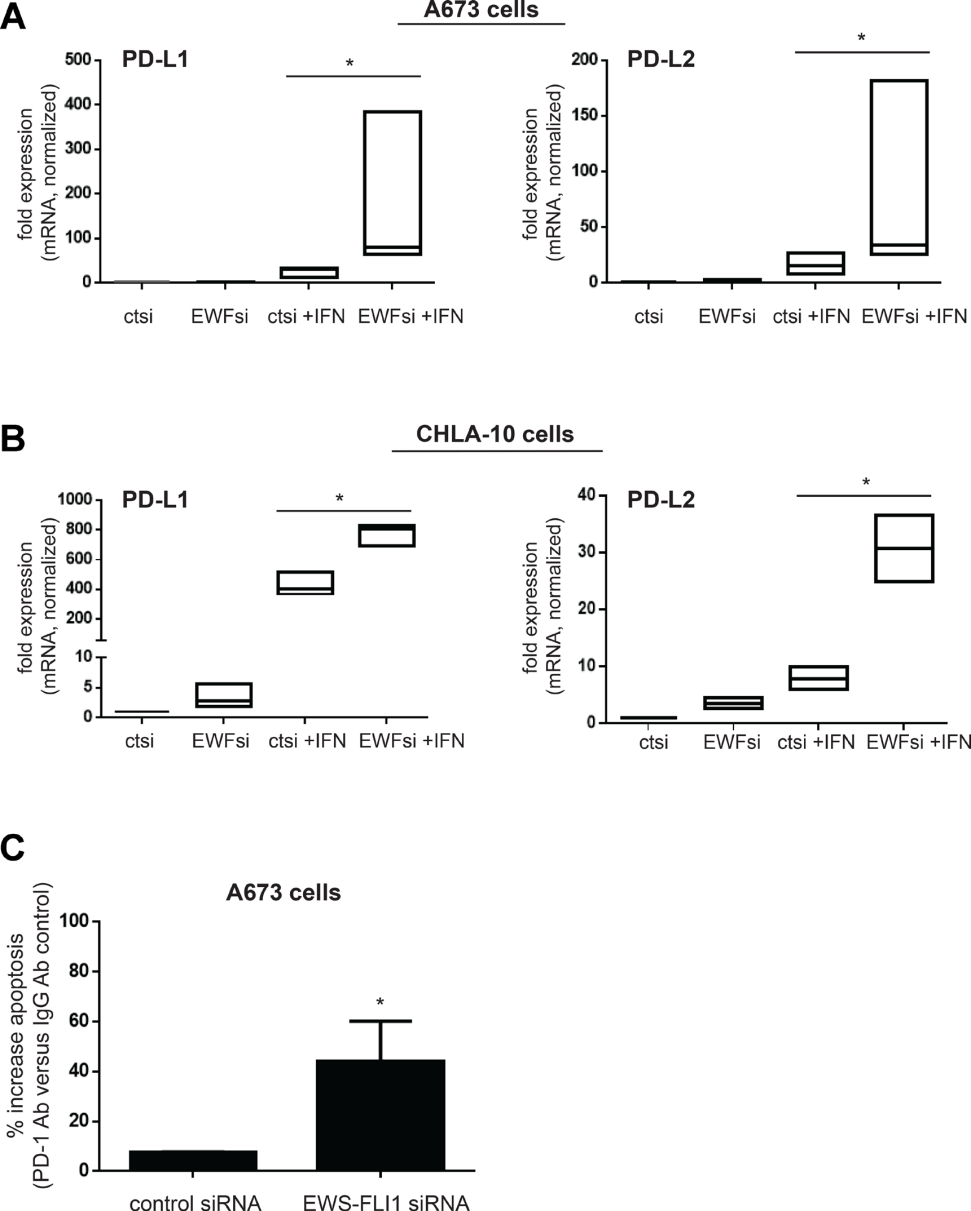
Lower EWS-FLI1 level results in upregulation of PD-L1 and PD-L2 in Ewing sarcoma tumor cells and blocking PD-1 increases T-cell mediated tumor cell apoptosis. (**A**, **B**) A673 (A) and CHLA-10 (B) Ewing cells were treated with control (ctsi) or EWS-FLI1 siRNA (EWFsi) and then treated ± IFN-γ for 24 hours. *CD274* (PD-L1) and *PDCD1LG2* (PD-L2) expression was analyzed by RT-PCR. (**C**) A673 cells treated with control or EWS-FLI1 siRNA were co-cultured with activated T-cells in the presence of IgG or PD-1 blocking antibody. Apoptosis was measured using an IncuCyte caspase assay and % increase in apoptosis with PD-1 versus IgG control antibody treatment is plotted for both control and EWS-FLI1 siRNA cells, ^*^p < 0.05.

We next wanted to investigate possible mechanisms by which lower EWS-FLI1 level causes resistance to T-cell mediated Ewing tumor cell apoptosis. Since we found that EWS-FLI1 low cells demonstrate an enhanced transcriptional response to IFN-γ, we chose to examine the expression of PD-L1 (programmed death-ligand 1) and PD-L2 (programmed cell death 1 ligand 2), both negative regulators of T-cell function whose transcription can be induced by IFN-γ [30]. In contrast to the positive influence of tumor ICAM-1 expression on T-cell activity, tumor cell PD-L1 and PD-L2 expression reduce T-cell driven anti-tumor cytotoxicity by binding to the PD-1 immune checkpoint receptor expressed on the surface of T-cells [31–33]. We found that following IFN-γ treatment, both *CD274* (PD-L1) and *PDCD1LG2* (PD-L2) mRNA is upregulated significantly more in EWS-FLI1 low cells compared to EWS-FLI1 high cells (Figure 6A, 6B). PD-L1 and PD-L2 surface protein expression is also higher in EWS-FLI1 low cells (Supplementary Figure 5).

**Figure 7:**
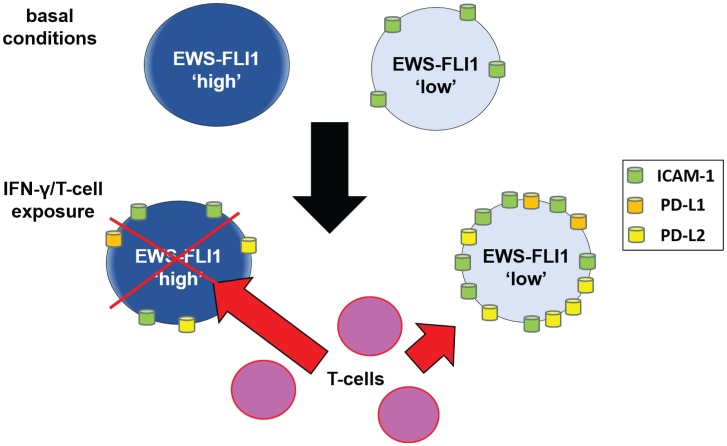
Schematic: proposed mechanism by which cells in the EWS-FLI1 low state demonstrate reduced sensitivity to T-cell mediated tumor cell apoptosis. Under basal conditions, EWS-FLI1 ‘low’ cells express significant ICAM-1. Upon IFN-γ/T-cell exposure, ICAM-1, PD-L1 and PD-L2 are upregulated significantly more in EWS-FLI1 low cells compared to high cells. With higher expression of PD-L1 and PD-L2, EWS-FLI1 low cells are more resistant to T-cell mediated tumor cell apoptosis. Red “X” represents tumor cell apoptosis.

We thus examined the impact of PD-1 blockade on the susceptibility of EWS-FLI1 high and low tumor cell susceptibility to T-cell mediated apoptosis and found that PD-1 blockade enhances T-cell mediated tumor cell apoptosis to a greater degree for the EWS-FLI1 low cells (Figure 6C). Together, these results suggest that despite having high ICAM-1 expression, EWS-FLI1 ‘low’ cells are able to evade T-cell mediated apoptosis by simultaneously upregulating factors that negatively impact the T-cell anti-tumor response, such as PD-L1 and PD-L2 (Figure 7).

## DISCUSSION

Metastatic and relapsed Ewing sarcoma is an often fatal disease, and advancing our understanding of the nuanced mechanisms that drive the survival of these tumor cells is essential. Very little is known about the Ewing sarcoma immune-microenvironment either at the time of diagnosis or at relapse. This gap in knowledge is due in large part to the fact that despite great effort by many laboratories around the world, no transgenic/syngeneic mouse model for Ewing sarcoma is currently available [12]. The current data available from analysis of human Ewing tumors reflects the fact that as like most pediatric tumors, the immune infiltrates in Ewing sarcomas are not as substantial as compared to adult carcinomas [7, 34]. However, of note, the proportion of CD8+ T-cells is higher in Ewing tumors compared to infiltrates of surrounding stroma [35], suggesting that the T-cell: tumor cell interaction could be important to examine in this cancer. Further, analysis of pre-treatment Ewing tumor specimens does not reflect the increase in tumor immune infiltration that could be seen upon treatment with radiation or chemotherapy nor the potential interactions of circulating tumor cells (CTCs) with immune cells [8, 10]. Because several recent reports have demonstrated that EWS-FLI1 expression level greatly influences Ewing sarcoma tumor cell behavior [13, 14, 16], and that EWS-FLI1 expression level within individual tumor cells is highly variable [14], we sought to examine how tumor cell EWS-FLI1 level impacts the response to and interaction with T-cells.

Because EWS-FLI1 low cells express more basal ICAM-1 and tumor ICAM-1 can enhance tumor cell: T-cell interaction and T-cell activation [19, 20], we hypothesized that EWS-FLI1 low cells would be more susceptible to T-cell mediated tumor cell killing as compared to tumor cells with higher EWS-FLI1 expression. In fact, we found the opposite, with cells in the EWS-FLI1 low state demonstrating less, not more, apoptosis when co-cultured with T-cells. Our studies indicate that EWS-FLI1 low cell immune resistance is due in part to the upregulation of PD-L1 and PD-L2, two potent negative regulators of T-cell function [33]. In general, the expression of PD-L1 and PD-L2 varies widely between cells within an individual tumor and between different tumor types. This is also true in Ewing sarcoma, with immunohistochemistry data demonstrating variable and often quite low PD-L1 expression [36]. Clinically, PD-L1 expression as low as 1–5% of cells can be associated with a significant response to PD-1 checkpoint inhibitors [37, 38] and this is the range of enhanced surface protein expression of PD-L1 and PD-L2 noted in the EWS-FLI1 low cells (Supplementary Figure 4). Individual patients with relapsed Ewing sarcoma have demonstrated a durable response to checkpoint inhibitor therapy, with one report of a patient with widely disseminated disease (lung and bone metastasis) achieve a complete radiological remission of greater than 6 months following initiation of pembrolizumab [3]. Published data has shown that EWS-FLI1 low cells typically represent only a minority of the cells within Ewing tumors (1–2% [14]) and we have now shown that EWS-FLI1 low cells can upregulate PD-L1 and PD-L2 expression and undergo enhanced T-cell mediated tumor cell apoptosis in response to blocking PD-1. Together these observations could suggest that cells in the EWS-FLI1 low state, which make up only a small fraction of tumor cells, would be particularly sensitive to checkpoint inhibitors. Thus, in most cases, a single agent checkpoint inhibitor treatment approach is unlikely to control bulk disease. Indeed, the inability for single agent PD-1 inhibitors to control bulk disease in Ewing sarcoma is reflected in results from the recent SARC-028 clinical trial [39].

So what might be a future role be for PD-1 blockade in Ewing sarcoma? The EWS-FLI1 low cell state has been shown to represent a minor, yet highly invasive subpopulation of cells within Ewing tumors [13, 14, 16]. We now have shown that the EWS-FLI1 low state also favors cell PD-L1/-L2 expression both at baseline and in response to IFN-γ stimulation. Perhaps PD-1 inhibitors could be used in combination with conventional chemotherapy in order to simultaneously target both bulk/EWS-FLI1 high tumor cells, which are more proliferative, and the minority population of invasive cells in the EWS-FLI1 low state, which would be expected to express PD-L1/-L2. As we have made little progress in improving outcomes for patients with metastatic Ewing sarcoma using various combinations of cytotoxic chemotherapy, looking toward incorporating immunotherapy into relapsed and metastatic Ewing sarcoma therapy is a logical next step in attempting to improve patient outcomes. The lack of a syngeneic/transgenic Ewing sarcoma mouse model makes immune-based Ewing sarcoma preclinical work challenging; but our ongoing, *in vitro* work continues to examine differences in immunologic behavior between Ewing tumor subpopulations and to provide insight into how to best harness emerging immunotherapies into novel treatment approaches for metastatic and relapsed Ewing sarcoma.

## MATERIALS AND METHODS

### Cell culture

Human patient-derived Ewing sarcoma cell lines were obtained from ATCC (A673, SK-N-MC), and the Children’s Oncology Group Childhood Cancer Repository (CHLA10). shA673-1c cells (with shRNA mediated tet-repressible EWS-FLI1 expression) are a kind gift from Dr. Olivier Delattre [14, 27]. Cells were cultured in RPMI plus 1% L-glutamine, 10% FBS and penicillin/streptomycin (P/S) (A673), IMDM plus 20% FBS, 1%ITS, 1% L-gluatmine and 1% P/S (CHLA10) or DMEM plus 10%FBS and 1% L-glutamine (SK-N-MC). shA673-1c cells were cultured in phenol-free RMPI with 10% tet-free FBS and 1% L-glutamine. shA673-1c cells were treated with 1 microgram/mL doxycycline or DMSO (control) in experiments. To passage, cells were detached from the plate using 0.05% trypsin (Gibco, cat #25300-062). Cell line identity was verified by StR profiling (University of Arizona Genetics Core). Cells were maintained in culture at 37 degrees C and 5% CO_2_. Routine testing was performed for mycoplasma using MycoAlert Detection Kit (Lonza, cat #LT07-318).

For IFN-γ treatment, Ewing tumor cells were placed in normal growth media with recombinant IFN-γ (R&D, cat #285IF100) at a concentration of 500 U/mL for the times indicated in individual experiments. For IFN-γ blocking experiments, IFN-γ neutralizing antibody (R&D cat #MAB2851-100) was used at a concentration of 1.5 micrograms/mL in serum free media.

### Isolation and activation of human T-cells

Human T-cells were isolated from the spare buffy coat of random, de-identified blood donors under an exemption from the University of Pittsburgh IRB (PRO17090430). Buffy coat was processed as previously described [40]. T-cells were isolated from the buffy coat using a commercially available negative selection T-cell isolation kit (STEMCELL, Easy Sep T-cell Isolation Kit cat# 19051, DNase I solution cat#07900). Isolated T-cells were placed in T-cell expansion media (STEMCELL, cat# 10981, Immunocult Expansion media) and activated over 72 hours using a CD2/CD3/CD28 antibody cocktail (STEMCELL, cat #10970).

### ELISA (enzyme-linked immunosorbant assay)

ELISA kits for IFN-γ (cat #DIF50) and sICAM-1 (cat #P161289) were purchased from R&D (Minneapolis, MN, USA). Cells were placed in 0% FBS containing media, treated as described in individual experiments, and the resulting supernatant was collected. Supernatants were spun at 1000 rpm at 4 degrees C for 5 minutes in order to eliminate any cell debris from the samples.

### RT-PCR

RNA isolations were performed using Qiagen Mini RNA isolation kit (Qiagen, #74136) for >500k cells or Qiagen micro RNA isolation kit (Qiagen, #74034) for <= 500k cells and RNA concentration was measured via NanoDrop (Thermo Scientific, Waltham, MA, USA).

cDNA synthesis was done using high capacity cDNA reverse Transcription kit (Applied Biosystems cat #4368813) and AppliedBiosystems Veriti 96-well thermocycler.

Q-PCR analysis was performed using TaqMan probes (ThermoFisher Scientific: EWS-FLI1 Hs03024497_ft, GAPDH Hs02786624_g1, RPLP0 Hs00420895_gH, ICAM-1 Hs00164932_m1, PD-L1 Hs00204257_m1, PD-L2 Hs00228839), Taqman Universal PCR Master Mix (Applied Biosystems, cat #4304437) and StepOnePlus Real-Time PCR system. RT-PCR experiments were conducted at a minimum in biological triplicates, with technical triplicates performed for each sample in an experiment.

### siRNA

Cells were transfected with control or EWS-FLI1 siRNA using RNAiMAx (Invitrogen, cat #56532) and OptiMEM (Gibco, cat #11058-021). Custom order siRNA using sequences previously validated to target the type 1 EWS-FLI1 fusion [22, 23] was obtained from Sigma Aldrich (5′–3′ direction): GGGUUCUGCUGCCCGUA GCU and AGCUACGGGCAGCAGAACCC. Control siRNA was also purchased from Sigma Aldrich (5′–3′ direction): GCCUUCAGCUGACGUCGACU and AGUCG ACGUCAGCUGAAGGC. For the shA673-1c cell system, see the cell culture section above.

### Flow cytometry

A BDBiosciences LSRFortessa (BD Biosciences, San Jose, CA, USA) flow cytometer with 355, 405, 488, 561, 640 nm lasers was used for all flow cytometry experiments. Cells requiring plate detachment prior to analysis were treated with Accutase (Invitrogen, #00-4555-56). Cells were analyzed using live/dead Near IR or live/dead aqua staining to establish live cell gating. Conjugated antibodies used for analysis included ICAM1-APC (Invitrogen cat # L10119, 17-0549-42, L34957), IgG1 isotype control, PD-L1 PE (R&D cat#FAB1561P) and PD-L2 PE (Invitrogen cat #12-5888-42). Raw data files were analyzed using FlowJo software (Ashland, OR, USA).

### IncuCyte apoptosis assays

Cancer cells were seeded in 100 uL of fluorobrite media (gibco, #A18967-01) + 2% FBS + 1%L-Glutamine in 96-well plate format and allowed to adhere to plate at 37° C, 5% CO_2_ for 8 h. Prior to loading into Incucyte, 100 uL additional volume was then added to each well in the form of solutions prepared in fluorobrite media containing caspase reagent and an appropriate number of activated T-cells in fluorobrite media were added to achieve the desired T-cell:cancer ratio as described in individual experiments. For all experiments, the final total volume per well was 200 uL and the final dilution of Caspase Reagent (Essen BioScience, Ann Arbor, MI, USA, #4440) was 1:2000. Cells were incubated and imaged at 2–3 hour intervals using an IncuCyte (Essen BioScience) for a maximum of 72 hours to avoid caspase reagent photobleaching. PD-1 and ICAM-1 blocking apoptosis experiments used anti-human ICAM1 (EMD Millipore, #MAB2146Z), anti-human PD-1 (R&D cat #ICA1017101) or IgG (#AB-108-C) antibody to the mixtures detailed above. IncuCyte experiments included ‘no T-cell’ controls to monitor for differences in baseline proliferation and apoptosis in the experimental groups. +T-cell conditions were normalized to no T-cell control conditions to determine fold-increase in apoptosis due to immune-killing.

### Western blot

Lysates of Ewing sarcoma cells were prepared as previously described [41]. The total protein concentration of the lysate was determined using Peirce BCA protein assay reagents (ThermoFisher Scientific, cat #23223, #23224) and a BSA standard curve (Thermo Scientific, #23208). 50 micrograms of protein was diluted in 4x loading buffer and run on a 4–15% precast SDS-PAGE gel (BioRad, #456-1084), then transferred onto a 2 micron nitrocellulose membrane (Biorad, #162112) at 95V for 50 minutes, and the membrane was then blocked in 5% nonfat dry milk/TBST (TBS + 0.05% Tween 20) for 1 hour at room temperature. Primary antibody [FLI1 (abcam #133485) 1:2000; Vinculin (Cell Signaling, #3901S) 1:1000] was then incubated overnight, rocking at 4C. Following washing (3× for 5 minutes in TBST), secondary antibody anti-Rb-HRP Promega, CAT#W401B) at a 1:10,000 dilution in TBST was incubated for 1 hour at room temperature. Following washes (3× for 5 minutes in TBST), the membrane was incubated in ECL2 (Thermo Scientific, #80196) for 5 min before film development. Band size was determined by comparison with a protein ladder (BioRad, #161-0374).

### Promoter reporter assays

The LightSwitch Luciferase Assay Kit (#32031), GoClone human *ICAM1* promoter reporter (S708388), LightSwitch positive control RPL10 promoter (#32006) and negative control LightSwitch random promoter (#32006) were purchased from Active Motif (Carlsbad, CA, USA) and used in experiments according to the manufacturer’s instructions.

### Statistical analysis

Experiments were conducted at a minimum in biological triplicates per cell line (see figure legends for the *n* per individual cell line). For flow cytometry experiments, a minimum of 10,000 events were analyzed per condition. MFI refers to the median fluorescence intensity. For siRNA experiments, it was pre-established that cell samples showing inadequate loss of expression (upon expression check) would be excluded from subsequent analyses. Statistical analyses were performed using GraphPad Prism version 6.0 (GraphPad Software). Error bars represent SD. *p* values are denoted in the figures using an asterisk and defined in the corresponding figure legend.

## SUPPLEMENTARY MATERIALS



## Abbreviations

CTC: circulating tumor cell
IFN-γ: interferon-gamma
MFI: median fluorescence intensity
