# Supplementary material for: EWS-FLI1 low Ewing sarcoma cells demonstrate decreased susceptibility to T-cell-mediated tumor cell apoptosis
Source: Oncotarget. 2019 May 21;10(36):3385–99. doi: 10.18632/oncotarget.26939 (PMC6534359; doi:10.18632/oncotarget.26939)
Supplement: Supplementary file 1 [file oncotarget-10-3385-s001.pdf]

EWS-FLI1 low Ewing sarcoma cells demonstrate decreased susceptibility to T-cell-mediated tumor cell apoptosis

SUPPLEMENTARY MATERIALS

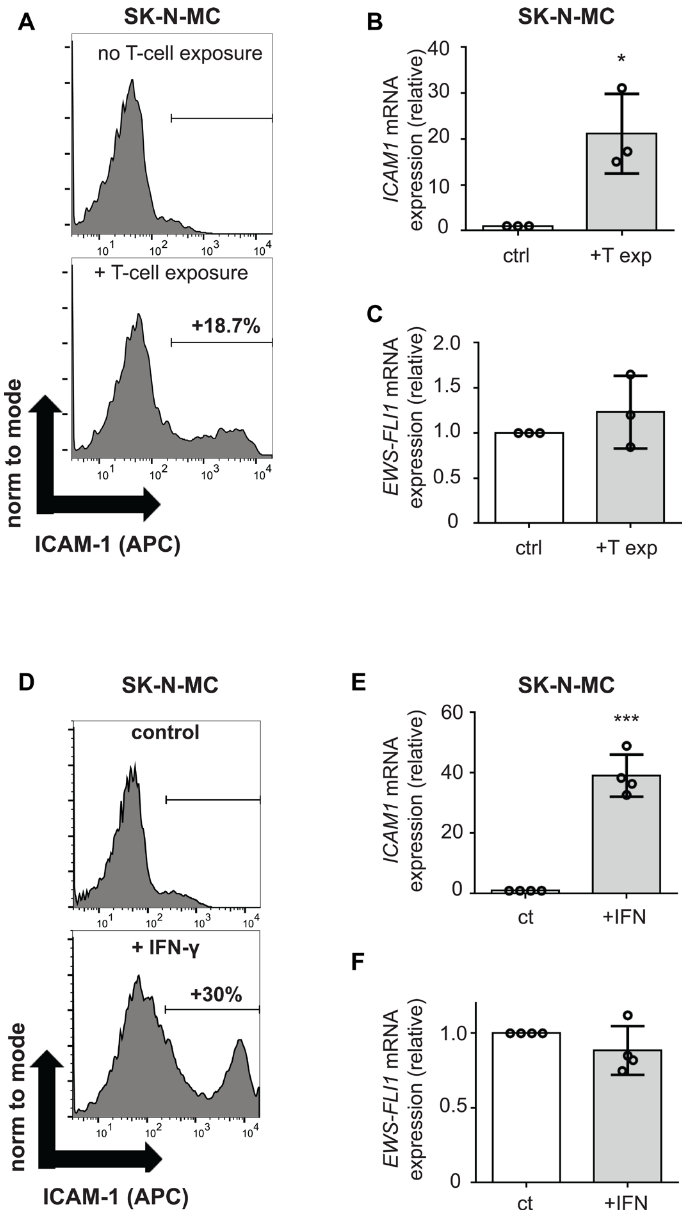

Supplementary Figure 1: Key experiments in a third Ewing sarcoma cell line, SK-N-MC. (**A**) SK-N-MC cells were co-cultured ± activated T-cells at a ratio of 1 T-cell per 50 tumor cells for 24 hours. Following incubation, T-cells were washed away and tumor cells were stained and analyzed for ICAM-1 surface expression. Graphs demonstrate live singlet cell populations. % denotes the frequency of ICAM-1+ cells upon analysis of a minimum of 10,000 total events. (**B**, **C**) RNA was extracted from tumor cells and corresponding cDNA was probed for changes in *ICAM1* (B) and *EWS-FLI1* (C) expression using RT-PCR (n = 3). Error bars represent SD. $^{*}p < 0.05$. (**D**–**F**) SK-N-MC cells were treated with 500 U/mL IFN-γ (+IFN) or vehicle control (ct) for 48 hours followed by analysis for surface ICAM-1 by flow cytometry (D) or RNA isolation and analysis for *ICAM1* (E) and *EWS-FLI1* (F) expression by RT-PCR (n = 4). ct/+IFN cell groups were compared using an unpaired t-test. Error bars represent SD. $^{***}p < 0.001$. Graphs in (D) demonstrate live, singlet cell populations. % denotes the frequency of ICAM-1+ cells upon analysis of a minimum of 10,000 total events. Circles on bar graphs in B–C and E–F indicate values for individual replicates.

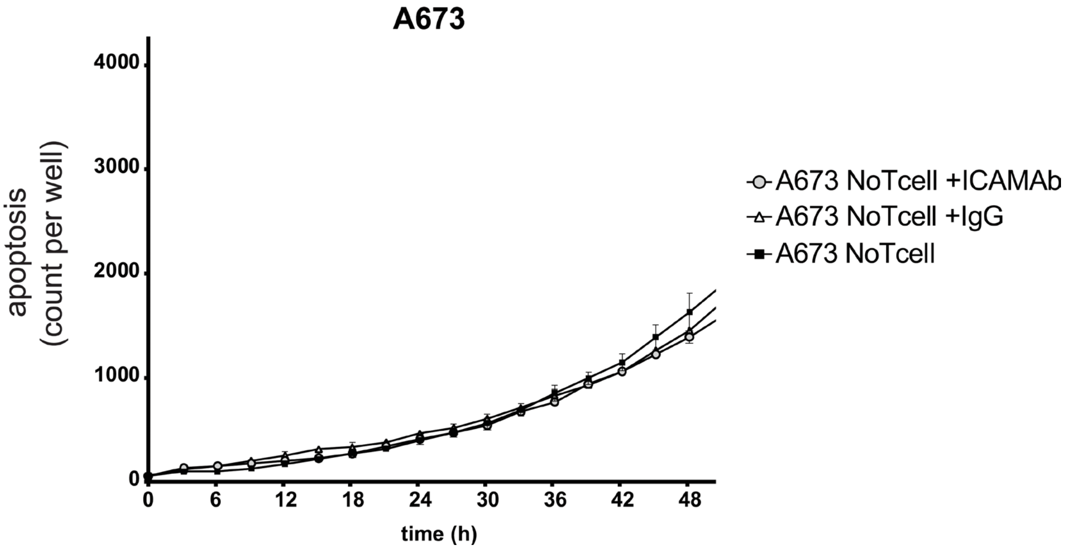

Supplementary Figure 2: In the absence of T-cells, ICAM-1 blocking antibody has no impact on tumor cell apoptosis. A673 Ewing sarcoma cells were incubated with ICAM-1 antibody, IgG antibody or no antibody controls in the absence of T-cells. Cells were monitored over time for apoptosis using an IncuCyte apoptosis assay. No significant difference in tumor cell apoptosis was noted between the groups in the absence of T-cells.

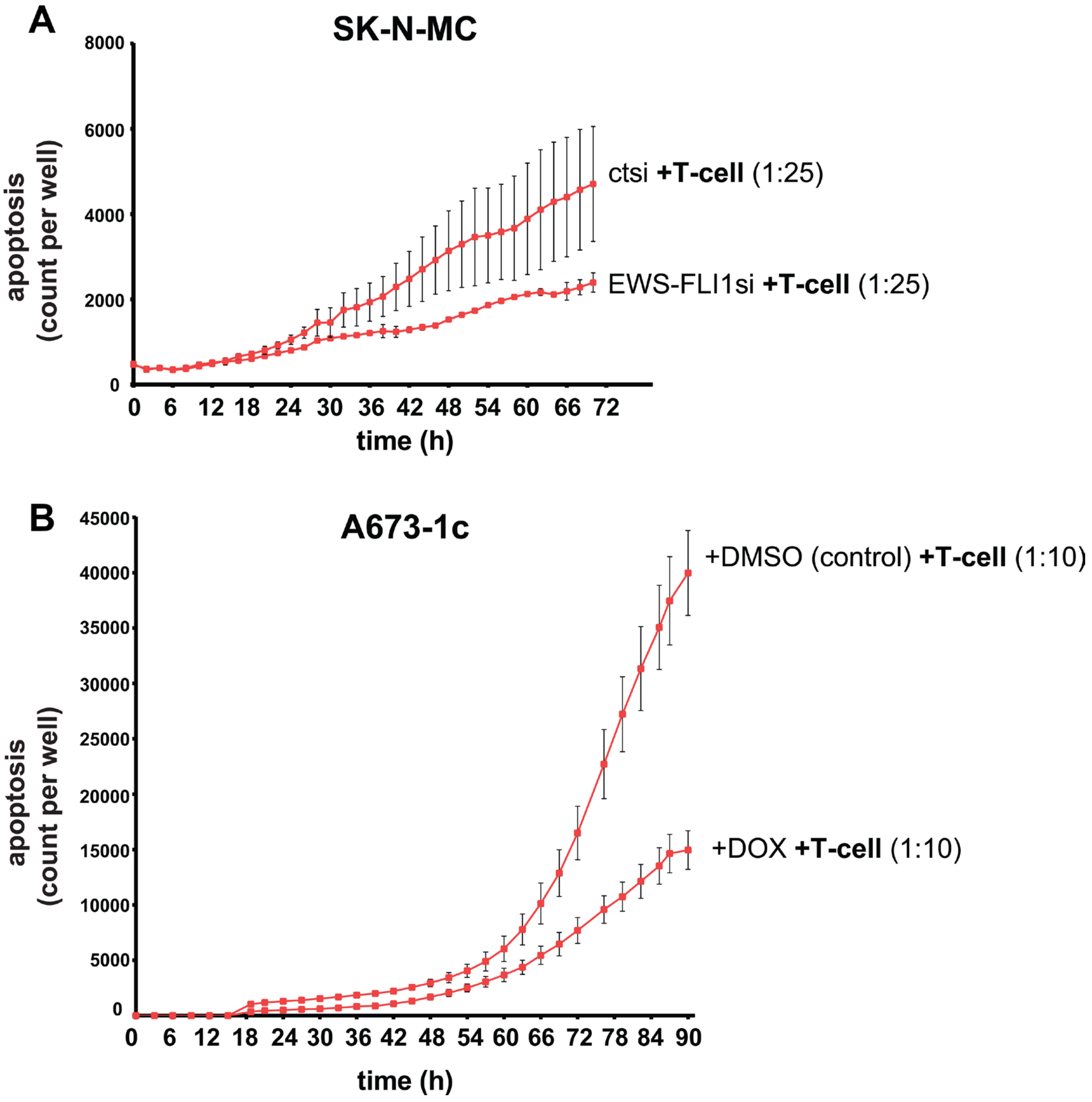

Supplementary Figure 3: Additional Ewing cell line and shRNA model system also demonstrate that the EWS-FLI1 low state results in decreased T-cell mediated tumor cell apoptosis. (**A**) SK-N-MC (*n* = 3) cells treated with control (ctsi) or EWS-FLI1 siRNA were placed in the in the presence (+T-cell) of activated T-cells at a ratio of 1:25 (T-cell : tumor cell) and monitored for tumor cell apoptosis using an Incucyte apoptosis assay. (**B**) shA673-1c cells were treated with DMSO or doxycycline (DOX) for 48 hours. DMSO control or DOX treated cells were then co-cultured with activated T-cells at a ratio of 1:10 starting at hour 18 and subjected to an Incucyte apoptosis assay. Error bars represent SD.

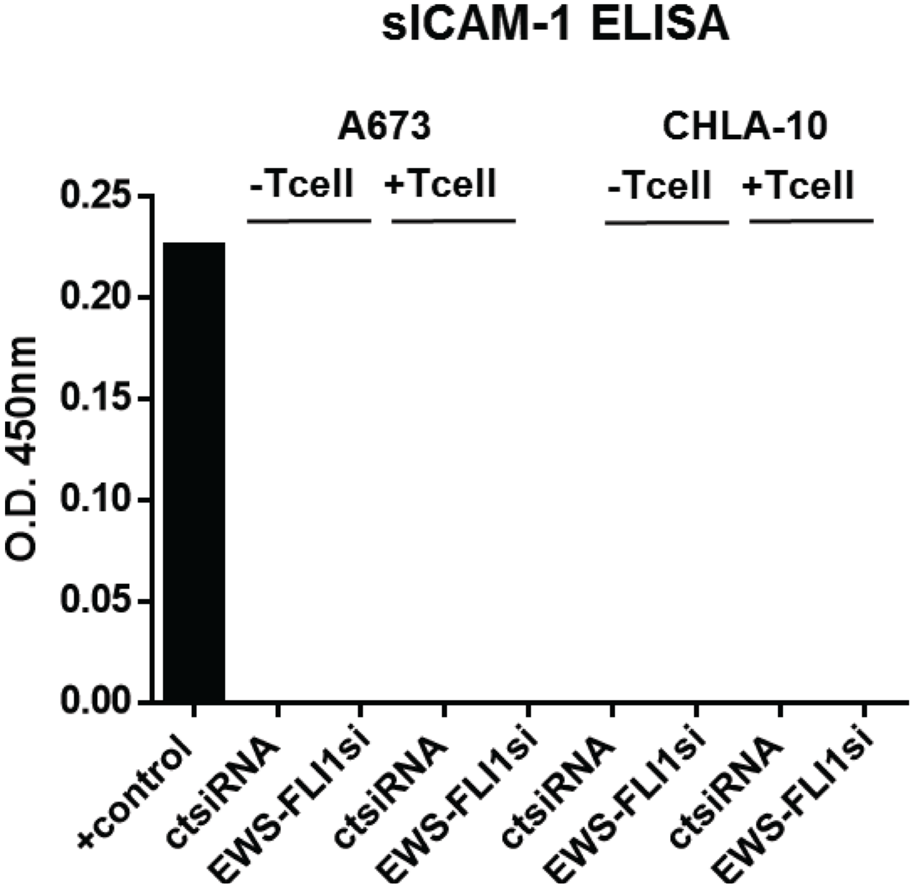

Supplementary Figure 4: EWS-FLI1 siRNA treatment does not induce the secretion of soluble ICAM-1. Conditioned media from A673 (n = 3) and CHLA10 (n = 3) cells treated with control (ctsiRNA) or EWS-FLI1 siRNA (EWS-FLI1si) ± T-cell exposure was subjected to a soluble ICAM-1 ELISA.

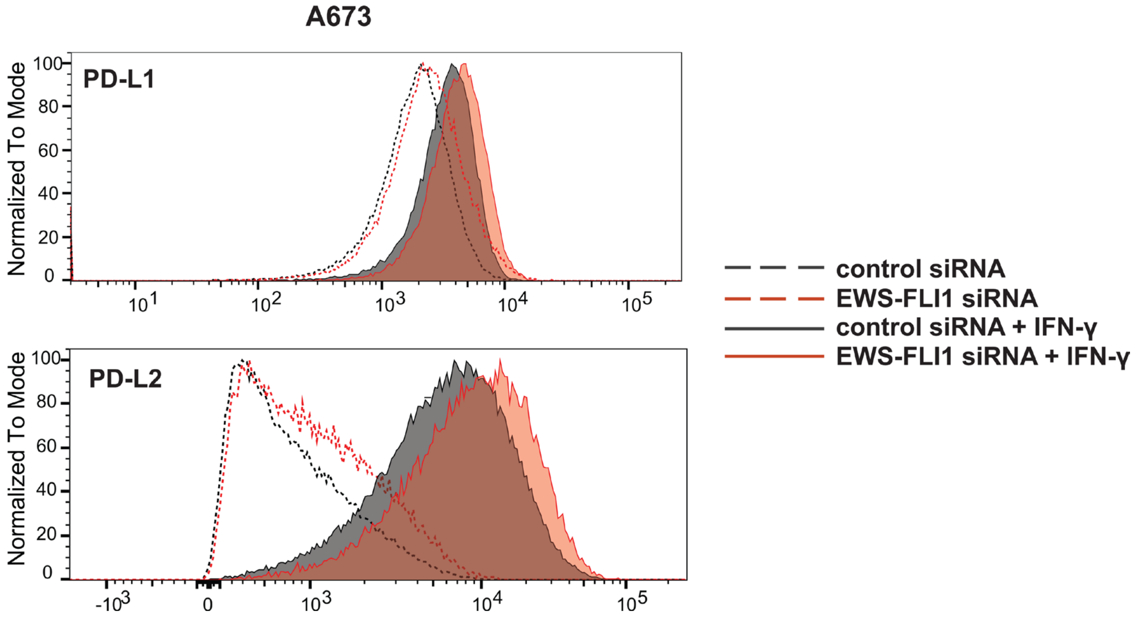

Supplementary Figure 5: IFNγ-induced PD-L1 and PD-L2 expression on the cell surface is greater in EWS-FLI1 low cells. A673 Ewing cells were treated with control (ctsi) or EWS-FLI1 siRNA (EWFsi) and then treated ± interferon-gamma (IFN) for 24 hours. Cells were then analyzed by flow cytometry for PD-L1 and PD-L2 surface expression. Experiments were performed in triplicate. Graphs demonstrate live, singlet cell populations.
